# Low-magnification narrow-band imaging for small gastric neoplasm detection on screening endoscopy

**DOI:** 10.1016/j.vgie.2022.04.007

**Published:** 2022-07-21

**Authors:** Ryuichi Nagashima

**Affiliations:** Nagashima Clinic, Yamagata, Japan

**Keywords:** DL, demarcation line, HM-NBI, high-magnification narrow-band imaging, iMS, irregular microsurface, iMV, irregular microvascular, K-T classification, Kimura-Takemoto classification, LM-NBI, low-magnification narrow-band imaging, NBI, narrow-band imaging, M-NBI, magnifying narrow-band imaging, rVC, revised Vienna classification of gastrointestinal epithelial neoplasia, WLI, white-light imaging

## Abstract

**Background and Aims:**

Microsurface patterns of the gastric mucosa can be observed using magnifying narrow-band imaging (M-NBI). However, the efficacy of M-NBI at low-magnification (LM-NBI) screening for detecting small gastric neoplasms is unclear.

**Methods:**

This prospective study was conducted at a single institution. LM-NBI, defined as minimal magnification that could reveal the microsurface pattern of the gastric mucosa, was performed after routine white-light imaging (WLI) observation of the stomach. Depending on the phase in which the neoplastic lesions were initially found, they were divided into the WLI group and the LM-NBI group, and the characteristics of these neoplastic lesions were investigated accordingly.

**Results:**

Sixty-five epithelial lesions (adenomas or noninvasive carcinomas) of 20 mm or less in diameter were identified in this study. Sixteen lesions were detected only with LM-NBI. Smaller lesions were detected using LM-NBI (*P* = .01). WLI took about 160 to 260 seconds, while LM-NBI required about 70 to 80 seconds. All lesions in the LM-NBI group had a background of map-like redness (n = 5) or atrophic/metaplastic mucosa (n = 11).

**Conclusions:**

LM-NBI was able to detect lesions overlooked by WLI, especially those in areas of map-like redness or atrophic/metaplastic mucosa of the stomach. Approximately one-quarter of newly diagnosed neoplasms were retrieved on routine examination during an extra 1.5 minutes.

Infection with *Helicobacter pylori* is the greatest risk factor for gastric cancer even after its eradication,^1^^,^^2^ and the early detection and accurate diagnosis of mucosal lesions is ideal for decreasing mortality rates. Therefore, endoscopic screening is vital in areas with a high incidence of *H pylori* infection.^3^

Many controversial criteria or algorithms have been used for the diagnosis and classification of gastric cancer on endoscopic examination, and the Japanese Gastroenterological Association recommends the magnifying endoscopy simple diagnostic algorithm for gastric cancer, which uses an evidence-based approach.^4^ In this algorithm, the first step is the identification of a suspicious lesion that is potentially a neoplasm under white-light imaging (WLI), for which magnifying endoscopy should follow. There must be 2 phases in the observation: the first is the screening phase to identify a suggestive lesion of neoplasm, and the second is the diagnostic phase to clarify the lesion’s detailed characteristics. Magnifying endoscopy and narrow-band imaging (NBI) are efficient for making accurate diagnoses,^5^, ^6^, ^7^ and magnifying narrow-band imaging (M-NBI) contributes accordingly. In M-NBI, irregular microsurface (iMS) patterns and irregular microvascular (iMV) patterns should be determined after identification of the demarcation line (DL) between the lesion and the background mucosa.^4^

Observing the color or morphological change is emphasized in the screening phase; however, it remains tacit knowledge to recognize a potential neoplasm under WLI, especially small lesions. Image-enhanced endoscopy, such as blue laser imaging-bright and linked color imaging, was recently reported as superior to WLI for discovering neoplasms within the stomach.^8^^,^^9^ Endoscopic screening revolves around image-enhanced endoscopy; however, NBI endoscopy cannot increase gastric cancer detection^10^ despite the use of its second-generation equipment.^11^

There is no consensus about the whole stomach observation using M-NBI in screening examination because it is a time-consuming task. The DL and iMS can be detected even at low magnification, and using low magnification may save the observation time. However, there are few reports on the use of low-magnification NBI (LM-NBI). Therefore, this study aimed to evaluate the efficacy of LM-NBI in detecting small neoplasia within the stomach. LM-NBI was performed after WLI in screening endoscopic examinations, and the lesion characteristics were investigated accordingly.

## Methods

### Study design, patients, and endoscopic procedure

This prospective analysis was performed at a single institution (Nagashima Clinic) by an endoscopist with more than 30 years of endoscopy experience. This endoscopist had performed approximately 30,000 examinations over the last 20 years. Patients who visited the clinic for check-ups or routine follow-ups between April 2019 and June 2021 were included after providing informed consent. Patient demographics and clinical characteristics were derived from their clinical medical records and analyzed. All epithelial neoplastic lesions 20 mm or less in diameter in these patients were identified and recorded.

The video endoscopic system used in this study comprised a video processor (EVIS LUCERA ELITE CV-290; Olympus Medical Systems, Tokyo, Japan) and a light source (EVIS LUCERA ELITE CLV-290; Olympus Medical Systems). GIF-HQ290, GIF-H260Z, and GIF-H290EC (Olympus Medical Systems) were used.

The endoscopic examinations were routinely performed via the oral route for observation of the pharynx, esophagus, stomach, and duodenum. If there was a request for sedation, 1% propofol was administered intravenously at an appropriate dose. To suppress peristalsis, 0.8% l-menthol was sprinkled into the stomach. After washing with Water-jet (Olympus Medical Systems), the gastric mucosa was first observed under WLI, followed by LM-NBI, which was defined as minimal magnification that could reveal the microsurface pattern of the gastric mucosa. If a lesion suggestive of a neoplasm was found, high-magnification NBI (HM-NBI), defined as that at a non-limited magnification, was conducted to clarify its detailed characteristics. After the lesions were measured, biopsies were performed in these patients if needed. The phase in which a suggestive lesion was initially found, WLI or LM-NBI, was recorded, and neoplastic lesions were divided into WLI and LM-NBI groups. All endoscopic examinations were recorded as fully moving images and still pictures with a clocking system (GT Finder; Medical Image Communication System, A-Z, Sendai, Japan). Forty pictures covering the entire stomach from distant to close-up views were taken during WLI, and 20 still images were recorded, mainly in close-up view in subsequent LM-NBI. Observation times were calculated individually, such as the time of the entire stomach, WLI-NBI, LM-NBI, and HM-NBI observations.

The presence of *H pylori* infection was determined using a ^13^C-urea breath test (UBit, Otsuka, Tokyo, Japan). Patient status was defined as follows: those who tested positive on the ^13^C-urea breath test were categorized as “pre-eradication”; those with a history of successful eradication were categorized as “post-eradication”; and those with no history of eradication with negative ^13^C-urea breath test result but atrophic changes in the gastric mucosa were categorized as “spontaneous eradication.”

### Endoscopic and pathologic criteria

Neoplastic lesions were categorized macroscopically using the Japanese Classification of Gastric Carcinoma provided by the Japanese Gastric Cancer Association,^12^ such as type 0-IIa, superficial elevated, type 0-IIb, superficial flat, and type 0-IIc, superficial depressed. According to the Kimura-Takemoto classification (K-T classification) of atrophic gastritis,^13^ atrophic status was evaluated as follows: closed-type atrophy was categorized as C-1, mild; C-2, moderate; and C-3, severe, and open type atrophy as O-1, mild; O-2, moderate; and O-3, severe. The map-like redness that is frequently observed after *H pylori* eradication is diagnosed using the Kyoto classification of gastritis.^14^ The vessel plus surface classification system^15^^,^^16^ was used under M-NBI.

Neoplastic lesions were pathologically diagnosed by application of the revised Vienna classification of gastrointestinal epithelial neoplasia (rVC)^17^ as follows: rVC 3, low-grade adenoma/dysplasia; rVC 4.1, high-grade adenoma/dysplasia; and rVC4.2, noninvasive carcinoma.

### Statistical analysis

The demographics of the study participants in the WLI and LM-NBI groups were compared using the Mann-Whitney U test for age and observation time and Fisher exact probability test for frequency data.

## Results

Sixty-five epithelial neoplastic lesions 20 mm or smaller were found during the study period, of which 16 were noted on LM-NBI observation. No poorly differentiated carcinomas of commensurate size were detected, and all were adenomas or differentiated adenocarcinomas. All lesions revealed DL, iMV, and iMS patterns on M-NBI.

Table 1 shows the demographic characteristics of each group. All lesions were related to atrophic gastritis, and there were no statistically significant differences in *H pylori* status or degree of atrophic changes in the K-T classification. Smaller lesions were found during the LM-NBI phase than during the WLI phase (*P* = .01), and all low-grade adenomas (rVC3) were detected during WLI observation (*P* < .01). There were no significant changes in the other lesion characteristics, such as morphology, color, location, or background mucosa. LM-NBI took a median of approximately 1.5 minutes. For the lesions detected by WLI, more time was spent in WLI observation compared to LM-NBI (*P* < .01). However, for the lesions that were missed by WLI and detected by LM-NBI alone, it was noted that the time spent in LM-NBI observation was higher than the time spent in WLI observation (*P* = .16). Between the 2 groups, there were no statistically significant differences in age, sex, use of endoscopes, or sedation.

**Table 1:** Clinical characteristics of the lesions

Variable	LM-NBI group (n = 16)	WLI group (n = 49)	*P* value
Median age, y (IQR)	74 (67-76)	71 (68-76)	.71
Sex			.15
Men	12	26	
Women	4	23	
Endoscope			.32
GIF-H290EC	13	29	
GIF-HQ290	2	10	
GIF-H260Z	1	10	
Sedation			.36
Yes	9	35	
No	7	14	
*Helicobacter pylori* status			.58
Pre-eradication	2	14	
Post-eradication	7	18	
Spontaneous eradication	7	16	
Not tested	0	1	
Atrophic gastritis (K-T classification)			.60
C-3	0	1	
O-1	1	4	
O-2	4	6	
O-3	11	38	
Size, mm			.01
–5	12	18	
6-10	2	18	
11-15	0	11	
16-20	2	2	
Pathology (rVC)			<.01
Low-grade adenoma/dysplasia (3)	0	13	
High-grade adenoma/dysplasia (4.1)	13	17	
Noninvasive carcinoma (4.2)	3	19	
Location			.76
Fundus	0	3	
Body	5	11	
Angle	2	5	
Antrum	9	30	
Morphology			.22
Type 0-IIa	3	19	
Type 0-IIa+IIc	4	5	
Type 0-IIc	9	22	
Type 0-IIb	0	3	
Color			.43
Red	7	30	
White	9	18	
No change	0	1	
Background mucosa			.13
Map-like redness	5	5	
Atrophic/metaplastic	11	43	
Nonatrophic	0	1	
Median observation time, s (IQR)			
Whole stomach	313 (270-357)	394 (319-473)	.04
WLI	167 (133-186)	265 (174-314)	<.01
LM-NBI	86 (53-181)	74 (40-112)	.16
HM-NBI	60 (29-78)	50 (33-106)	.66

*HM-NBI*, High-magnification narrow-band imaging; *IQR*, irregular microvascular; *K-T classification*, Kimura-Takemoto classification; *LM-NBI*, low-magnification narrow-band imaging; *rVC*, revised Vienna classification of gastrointestinal epithelial neoplasia; *WLI*, white-light imaging.

**Figure 1:**
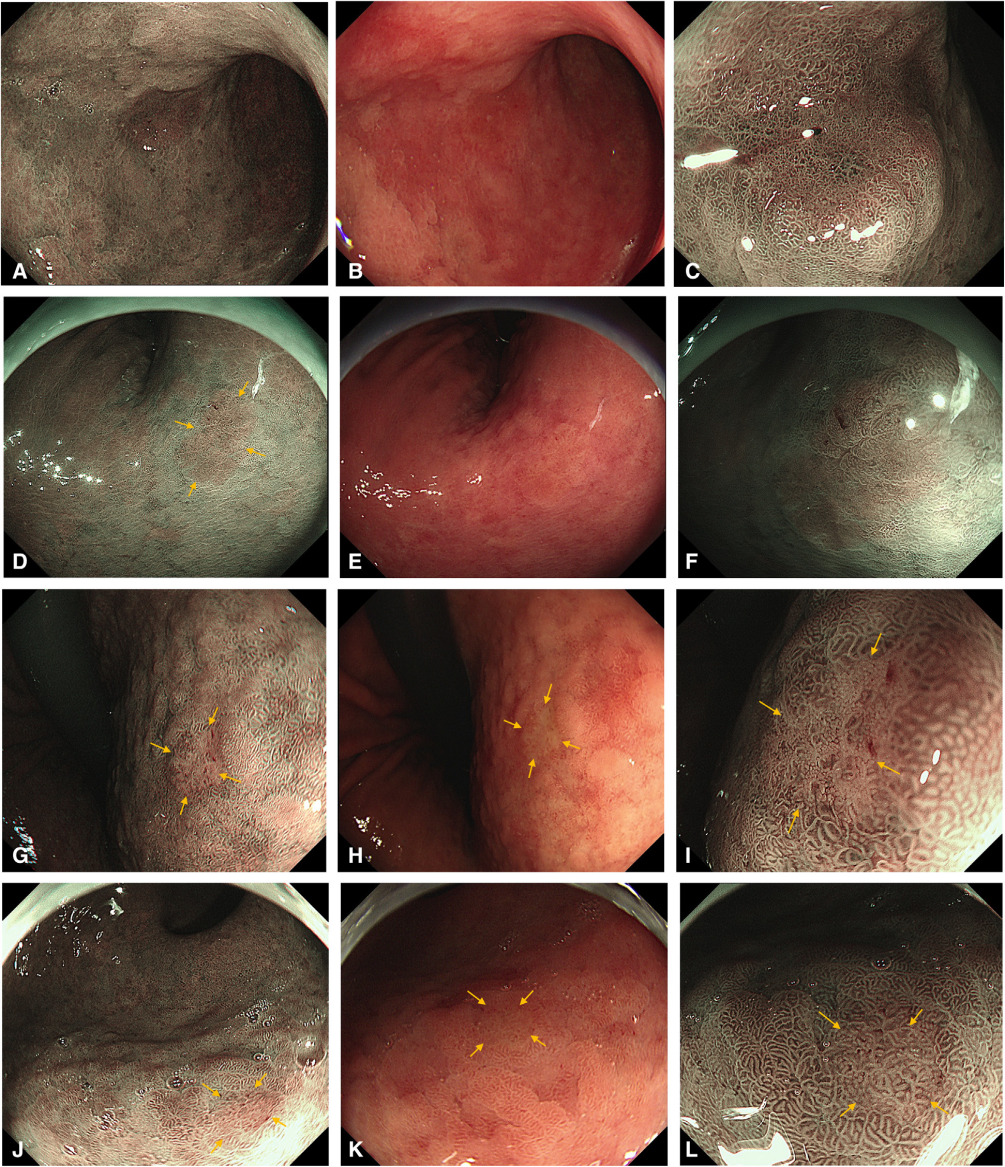
The left-end column reveals low-magnification narrow-band imaging (LM-NBI) observation on which the neoplastic lesions were found. The middle column shows white-light imaging (WLI) of almost the same views as LM-NBI. The right-end column is a high-magnification narrow-band imaging (HM-NBI) observation of the lesions. Case 1 is a noninvasive cancer. **A,** The brownish depressed lesion in the anterior wall of the lower body is visible. **B,** The lesion is reddish on WLI retrospectively and difficult to distinguish from the map-like redness in the background. **C,** The demarcation line (DL) is observed. **D,** The brownish superficial elevation is revealed for Case 2 (*arrows*); this is a noninvasive carcinoma. **E,** The lesion is vague on WLI. **F,** The DL is distinct on HM-NBI. **G,** The very small, depressed lesion can be seen in Case 3 (*arrows*); this is a high-grade adenoma. **H,** In WLI, the suggestive lesion is shown by *arrows*. **I,** HM-NBI visualized DL (*arrows*). **J,** In Case 4, a high-grade adenoma, a minute depression is indicated by *arrows*. **K,** WLI reveals the whitish depression retrospectively (*arrows*). **L,** The DL is shown by *arrows* on HM-NBI.

Sixteen lesions detected on only LM-NBI were further divided into 2 groups. Five lesions had the map-like redness with background mucosa (eg, Case 1 and 2 in Fig. 1 and Video 1 [available online at www.giejournal.org]), while 11 lesions were found in atrophic or metaplastic mucosa (eg, Case 3 and 4 in Fig. 1 and Video 1). The same lesion categories were found in the WLI phase (5 and 43 lesions for each); these lesions are compared in Table 2. Lesions in map-like redness were not very small, and there was no statistically significant difference between NBI and WLI in diameters (*P* = .12). Further, LM-NBI doubled the number of detected lesions from 5 to 10 in this situation. In atrophic or metaplastic mucosa, all lesions greater than 5 mm were found on WLI; however, approximately one-third of lesions (11/28) of 5 mm or smaller were overlooked on WLI (*P* < .01).

**Table 2:** Background mucosa and diameter of the lesions

Variable	LM-NBI group	WLI group	*P* value
In map-like redness	5	5	.12
–5 mm	1	0	
6-20 mm	4	5	
In atrophic/metaplastic mucosa	11	43	<.01
–5 mm	11	17	
6-20 mm	0	26	

*LM-NBI*, low-magnification narrow-band imaging; *WLI*, white-light imaging.

## Discussion

This clinical study demonstrated that LM-NBI used after WLI was able to detect lesions overlooked by WLI. The retrieved lesions were estimated to be approximately one-quarter of the newly diagnosed small gastric cancers/adenomas. This was accomplished by the addition of an extra 1.5 minutes during the routine examination. A screening observation using LM-NBI seems to be an efficient strategy for the early detection of neoplastic lesions, especially those in map-like redness or in atrophic/metaplastic mucosal areas of the stomach.

Atrophic gastritis and intestinal metaplasia caused by *H pylori* infection are well-known origins of gastric neoplasia. The map-like redness observed after successful eradication is reportedly a useful endoscopic marker for predicting gastric cancer.^18^ In both backgrounds, M-NBI had superior diagnostic efficacy compared to WLI,^19^^,^^20^ and thus the screening use of M-NBI seems quite logical. Lesions detected during LM-NBI for map-like redness were not very small. Hence, the cause of oversight may not be their size but rather their colors. It was difficult to distinguish these lesions on WLI; however, LM-NBI could visualize the neoplasms using DL and iMS. Lesions 5 mm or smaller in the atrophic/metaplastic mucosa were found during LM-NBI. The benefit of magnification was assumed in this situation.

There were some statistically significant differences in the observation time, although the reasons seem equivocal. More time was required for WLI observation in the WLI group than in the LM-NBI group. This does not necessarily indicate that the time invested resulted in more newly discovered neoplasms. When a lesion was found, the observation continued using the same method, and still images were increasingly taken. Consequently, the observation time was prolonged. Although the difference was not statistically significant, LM-NBI took longer in the LM-NBI group than in the WLI group.

Unfortunately, small diffuse-type carcinomas were not found in this study. Therefore, the potential of an additional LM-NBI procedure to detect these lesions is unclear. We could not expect much since M-NBI reportedly has limitations in the diagnosis of signet ring cell carcinoma.^21^ Diffuse-type carcinoma often arise not associated with chronic mucosal changes,^22^ and no lesions in the LM-NBI group appeared in the non-atrophic mucosa in this study. Therefore, screening using LM-NBI in the mucosa without atrophy may be nonessential.

The strengths and weaknesses of this study are related to the fact that it was performed by a single endoscopist in 1 clinic. Each endoscopic examination was performed evenly and methodically without bias. However, the generalization of these results may not be applicable. A future randomized study needs further consideration. The endoscopic examination cannot be accomplished only by LM-NBI because the fear of overlooking lesions in which WLI has superiority, such as signet ring cell carcinomas. The additional effect of LM-NBI is so obvious that the lesions of the LM-NBI group were hardly detectable using WLI observation only. Presumably, lectures based on endoscopist experience are required. In Yamagata City, where my clinic is located, endoscopic screening for gastric cancer is performed in alternate years. All participating endoscopists are required to attend annual lectures on screening endoscopy. I have already presented some of these lesions in such lectures. The effectiveness of the education should be evaluated individually.

Recently, artificial intelligence has made great progress in detecting precancerous conditions and early gastric cancer.^23^^,^^24^ The multiple and wide observation aspects of LM-NBI place some burden on the endoscopist, and missed diagnoses are possible. Artificial intelligence–based support systems may be compatible with these screening observations.

## Disclosure

*The author disclosed no financial relationships.*

## Supplementary data

Video 1 The moment in which the lesion was found under low-magnification narrow-band imaging (Cases 1 to 4 in Fig. 1). The demarcation line is recognized after the elusive color change is picked out. On following high-magnification narrow-band imaging, irregular microvascular pattern is observed in each video clip. Note that the lesions are hard to detect during white light imaging.
